# Low-Cost Inkjet Printing Technology for the Rapid Prototyping of Transducers

**DOI:** 10.3390/s17040748

**Published:** 2017-04-01

**Authors:** Bruno Andò, Salvatore Baglio, Adi R. Bulsara, Teresa Emery, Vincenzo Marletta, Antonio Pistorio

**Affiliations:** 1 DIEEI-University of Catania, v.le A. Doria, 6–95125 Catania, Italy; salvatore.baglio@unict.it (S.B.); vincenzo.marletta@dieei.unict.it (V.M.); antonio.pistorio@dieei.unict.it (A.P.); 2 Space and Naval Warfare Systems Center Pacific, Code 71000, San Diego, CA 92152-5000, USA; bulsara@spawar.navy.mil (A.R.B.); teresa.emery@navy.mil (T.E.)

**Keywords:** direct writing, printing techniques, inkjet printing, transducers, low cost

## Abstract

Recently, there has been an upsurge in efforts dedicated to developing low-cost flexible electronics by exploiting innovative materials and direct printing technologies. This interest is motivated by the need for low-cost mass-production, shapeable, and disposable devices, and the rapid prototyping of electronics and sensors. This review, following a short overview of main printing processes, reports examples of the development of flexible transducers through low-cost inkjet printing technology.

## 1. Introduction

Recently, the scientific community has shown a growing interest in the possibility of developing low-cost flexible electronics by exploiting innovative materials and printing technologies. This interest is driven by several reasons: (i) the need for low-cost mass-production processes e.g., RFID tags, antennas, keyboards, displays, and flexible sensors [1,2]; (ii)numerous applications requiring shapeable and disposable devices where low-cost and flexible sensors can be conveniently used; and (iii) the need for the quick realization of electronics and sensors. As an example, the harsh conditions often encountered in many applications, such as limited accessibility, presence of corrosive gases, and high daily and seasonal temperature changes, often pose severe constraints on the use of expensive devices. Moreover, the possibility to rapidly develop inexpensive devices and sensors is of great interest for the scientific community as a whole.

Among printing technologies two main classes can be identified: the first one includes all techniques requiring mask-based or photolithographic processes, e.g., screen printing and printed circuit board (PCB); the second class is related to direct printing technologies, e.g., inkjet Printing (IJP). A benchmark between PCB technology and IJP is useful to identify the optimal solution for the rapid development of low cost sensors and electronics [3]. This analysis highlights the point that low-cost inkjet printing shows limitations in terms of available materials, although it is suitable for the quick realization of devices. Conversely, PCB-based techniques are well suited for mass production, however, the development times and costs are not in line with the needs of fast prototyping, especially for research purposes. The same advantage/constraint applies to roll-to-roll lithography, which provides very high-resolution in patterning functional materials on flexible substrates, while the high attendant cost for low production rates makes this technology suitable for mass production [1].

In the following section an overview of the main printing processes is presented, with a specific focus on inkjet printing. Section 3 provides examples of transducers developed by low-cost inkjet printing technology, while concluding remarks are presented in Section 4; this section also highlights a benchmark between printing technologies.

## 2. Printing Processes

Among printing processes, screen printing and inkjet printing have received the most attention for the realization of sensors.

### 2.1. Screen Printing: A Short Overview

Screen printing is based on the use of masks and a pressure roller mechanism. Masks are used to delimit areas where the target material must be deposited on the substrate. A good range of conductive, insulating, and functional materials, compatible with screen printing, are now commercially available. Screen printing can be listed as a low-cost technology, with the peculiarity that thick layers can be realized, thus, conferring unique performances to the devices, e.g., by increasing a track’s conductivity, although at the expense of other device specifications, such as flexibility. The main drawbacks of screen printing technology stem from the waste of materials and the needs for masks, which make this approach unsuitable for the rapid prototyping of devices, especially compared to direct printing techniques. Many examples of transducers realized by screen printing are available in the literature. Among others, screen printing has been successfully used for the realization of ammonia sensors [4], different kinds of moisture sensors [5], and force sensors [6,7].

### 2.2. Inkjet Printing

Inkjet printing belongs to the wider class of direct writing techniques; here, direct writing includes all processes which can be used to deposit functional and/or structural materials onto a substrate in defined locations and patterns [8].

Different technologies, exploiting inkjet printing, have been developed to realize electrodes and functional layers. As an example, conductive inkjet technology is a mixed two-step process based on the inkjet printing of a catalytic ink through a roll-to-roll process, followed by an electro-less copper plating [9]. This process is good for large mass production and is unlimited in length thanks to the roll-to-roll process. However, the process is, also, time consuming and not easily accessible by end users.

In contrast to processes requiring masks and/or post-processing steps, off-the-shelf inkjet printing is a mask-less drop-on-demand technique, which allows for the direct writing of conductive or functional ink layers on flexible or rigid substrates, without the need for complicated post-processing. Savings of production time, lowered costs and wastage of materials, as well as high spatial resolution, good reproducibility and ease of scalability can be listed as the main advantages of inkjet printing; the cost of inks is considered the main drawback. On the above basis, it can be affirmed that inkjet printing affords the possibility of combining the performances of flexible substrates and functional inks with applications to the requirements for the quick development of sensors and electronic components [10,11,12,13,14,15,16,17,18,19,20,21,22,23,24,25,26,27,28,29,30,31,32]. This is a unique feature, which allows the designer to immediately realize and test the device and, eventually, to promptly repeat the design and testing loop. Conductive metal inks, resistive polymers, e.g., poly(3,4-ethylenedioxythiophene) polystyrene sulfonate (PEDOT-PSS), and functional polymers, e.g., polianiline-PANI, are widely used for the realization of sensors via inkjet printing [10,11,12,13]. Recently the possibility of printing carbon nanotube sensing layers has been also explored [17,25].

When considering printing equipment, several options are available on the market [14,15]. Professional inkjet printers are expensive due to the requirement for both printing heads to be compatible with different kinds of inks, e.g., metal and polymeric based inks, and the implementation of repeated printing cycles. These printers allow for the realization of complex devices in terms of topology and layer architecture. The compatibility of piezoelectric printers with different solvents and inks, make them the favored candidates for the rapid prototyping of electronic devices, compared to, e.g., thermal printers. The typical resolutions of piezoelectric printers are on the order of the tens of micrometers. Office inkjet printers, which can be used to realize simple devices, usually suffer from incompatibility with functional inks and, especially, with metal inks due to their viscosity and nozzle occlusion problems. Moreover, multilayer architectures are quite difficult to implement with low-cost printers. Nevertheless, it bears noting that, recently, many efforts have been dedicated to synthesizing conductive inks compatible with the printing heads found in low-cost office printers; this boosts their use for the rapid prototyping of lab-scale devices, especially for research and educational purposes [33,34,35,36].

We now list some realization and applications of inkjet printed sensors. As an example, low-cost strain sensors have been developed [16,17,18] through the use of transparent and conducting spray-deposited films of single-walled carbon nanotubes [17], interlocked arrays of high-aspect-ratio Pt-coated polymeric nanofibers on thin polydimethylsiloxane layers [18], as well as Metalon^®^ water-based silver ink printable by low-cost inkjet printers [19]. A capacitive accelerometer for the detection of the human pulse has been developed by direct-printing patterns of silver nano-inks on pre-patterned flexible paper substrates [20]. A low-cost and customizable accelerometer has been realized by implementing a resistive readout strategy through strain gauges printed on the four spring-legs holding a central PET (polyethylene terephthalate) membrane [21]. A mass is attached to the central membrane to improve the device responsivity. A polymeric actuator has been realized by joining a layer of poly-vinyledene fluoride trifluoroethylene and silver printed electrodes [22]; the device operates on the micro-scale and requires driving voltages on the order of hundreds of volts. Inkjet-printed actuators have been developed for positioning applications, with sensitivity on the order of hundreds of micrometers [23]. Fully inkjet-printed strain sensor have been used in telemetric sensing systems, adopting inductive coupling [24]. Carbon nanotubes (CNT) and copper nanoparticle inks have been used to develop strain gauges on flexible substrates [25]. A tin dioxide (SnO_2_) sensor has been fabricated by inkjet printing onto polyimide foil [26]. Inkjet printing has been used to realize a P3HT (poly(3-hexylthiophene) based photoactive strain sensor, exploiting PEDOT-based conductive thin films [27], as well as a skin-conformable temperature sensor [28]. A 3-D microfluidic channel and electromagnetic sensor have been used to develop an all-inkjet-printed microfluidic microwave sensor [29]. An electrochemical sensor has been realized by inkjet printing carbon nanotubes on paper [30]. An inkjet-printed interdigitated electrode, a gas sensitive ink and a dipole antenna for wireless sensing have been combined to develop a completely passive humidity or gas sensor [31]. An interesting field of application of inkjet printing technology are sensate media, which can be defined as a surface filled by a very large number of sensors that mimic biological skin [32]. The use of functionalized substrates, e.g., silane-based hydrophobic paper, is proposed to improve the lateral resolution of IJP techniques [37], while paper substrates have been used to develop inexpensive electronic devices, including sensors and actuators [38].

### 2.3. Hybrid Technology

To cope with the limitations of the above-listed printing techniques, mixed approaches for the realization of low-cost printed devices have been proposed in the literature [7,8,9]. Conductive structures, such as wires, coils, and capacitive electrodes, are usually implemented by screen printing technology. As an example of hybrid technology, the development of conductive layers by polymers, such as PEDOT-PSS, requires several printing cycles which are not implementable with low-cost inkjet printers. However, once electrodes have been realized, functional layers, e.g., for strain and gas sensing, are successively deposited by low-cost inkjet printers. The main drawbacks of hybrid approaches are usually related to the restrictions of screen printing technology; these have already been listed in the preceding sub-section.

Examples of devices realized through hybrid approaches are resistors, contacts, and electrodes by PEDOT-PSS on polyethylene terephthalate (PET) [7], all-polymer RC filters [39], PANI-based devices for the detection of ammonia [4], and complex MEMS structures with silver nanoparticles [9]. Hybrid processes, also exploiting printed circuit board technologies, have been used to realize accelerometers [40,41], as well as devices for energy harvesting [42].

## 3. Examples of Low-Cost Printed Devices

In spite of devices realized by high cost professional printing equipment, nowadays researchers are, also, investigating in low-cost solutions for the development of fully-printed sensors. This is a major area of investigation of the DIEEI laboratory of the University of Catania-Italy. The DIEEI research group is addressing the possibility of realizing inexpensive flexible sensors by exploiting low-cost inkjet solutions and, if required, simple post-processing with the main objective of lab-scale prototyping especially for research and educational purposes.

In the following, examples of printed transducers realized by an office printer followed by inexpensive post-processing are given; they have, mainly, been developed at the SensorLab, DIEEI, University of Catania, Italy. Some recent work has been carried out in collaboration with SPAWAR Systems Center Pacific, San Diego.

### 3.1. All-Inkjet Printed Strain Sensors

**Figure 1:**
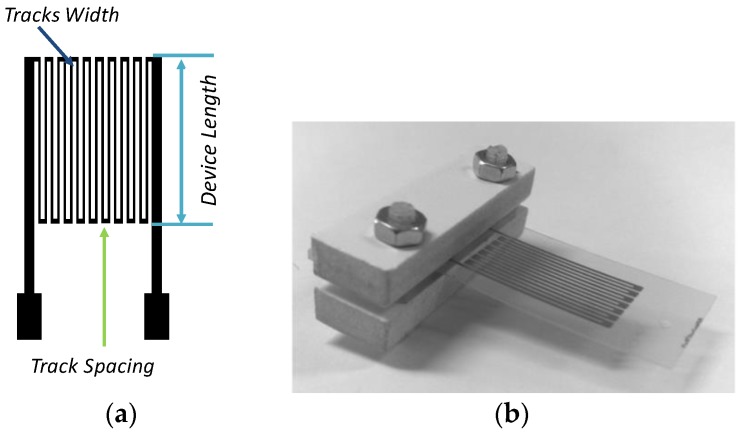
(**a**) Layout and (**b**) a real view of the strain sensor developed. The device length is 10 mm.

**Figure 2:**
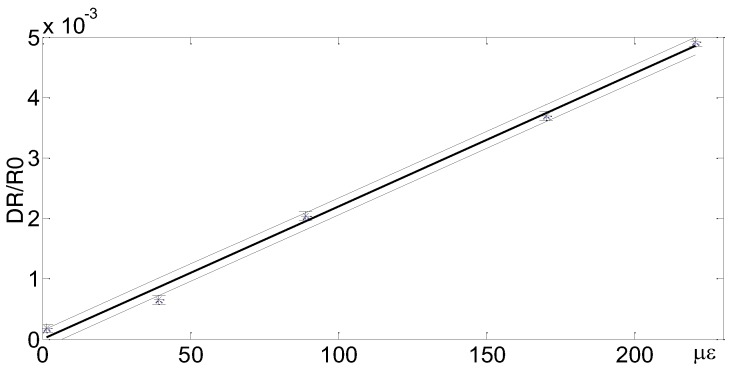
Response of the low-cost inkjet-printed strain sensors developed at the SensorLab@DIEEI of the University of Catania, Italy.

Figure 1 shows a typical layout and a real view of an inkjet-printed strain sensor [19]. The conductive pattern has been realized by inkjet printing a single layer of the silver nano-particle solution “Metalon^®^ JS-B15P”-Novacentrix (Austin, Texas) on a PET (poly-ethylene terephthalate) substrate, using a cheap EPSON piezo inkjet printer. The PET thickness is 100 μm, while track and spacing dimensions are 200 μm and 300 μm, respectively. The latter have been fixed by taking into account the constraints introduced by the low-cost inkjet printing technology. Electron microscopy (SEM) inspection of the electrodes highlights the limits of the printer in patterning the Metalon^®^ ink. A nominal printing resolution of 200 μm has been estimated. Actually, a track spacing of 200 μm is able to guarantee a suitable track separation, while bringing the track spacing to 150 μm could compromise the trace insulation. A similar analysis leads to a minimum allowed track width of 200 μm to guarantee the trace electrical continuity. The sensor response in terms of the relative variation of the sensor resistance as a function of the imposed strain is shown in Figure 2. The above results, which have been obtained by performing repeated experiments through a dedicated experimental setup, are in line with the expected behavior and show very good performance, in terms of the gage factor, as compared to the state of the art [19].

### 3.2. A Low-Cost Accelerometer Developed by Inkjet Printing Technology

**Figure 3:**
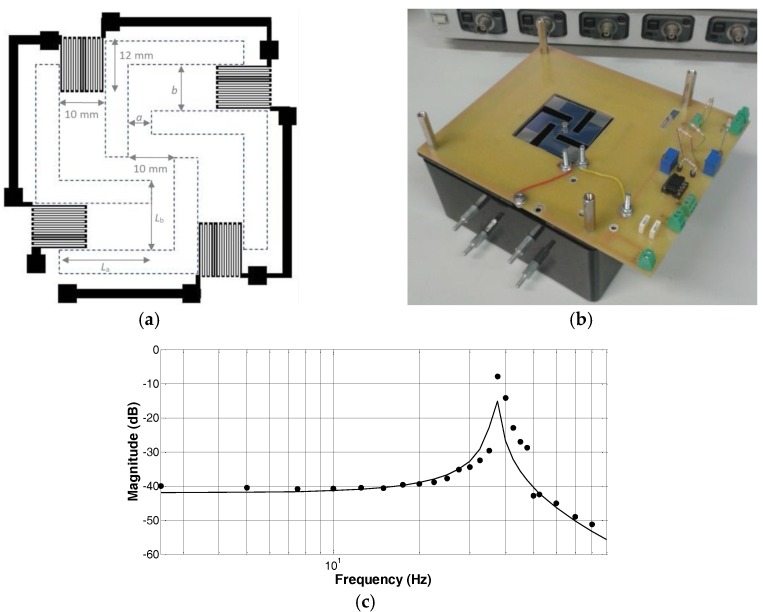

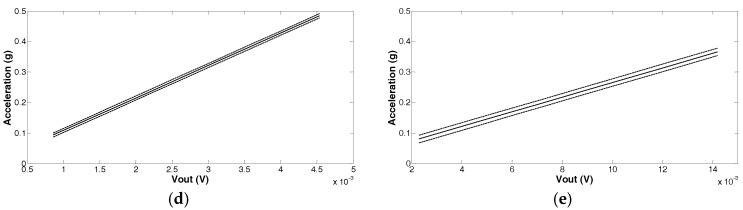
(**a**) Layout of the inkjet-printed accelerometer developed at the SensorLab@DIEEI of the University of Catania, Italy. Each strain gauge is about 12 mm long and 10 mm wide; the spacing and the track width are 300 µm, while the thickness of the conductive tracks is about 200 nm. The crab-leg beams have the following dimensions: a = 5 mm, b = 10 mm, L_a_ = 20 mm, and L_b_ = 15 mm for the thigh and the shin segments, respectively. The dashed lines represent the empty areas; (**b**) real view of the assembled final prototype of the developed inkjet-printed accelerometer; (**c**) frequency response of the device: experimental (black dots) and predicted (solid line); and (**d**,**e**) the calibration diagrams of the inkjet-printed accelerometer at 10 Hz and 35 Hz, respectively.

The layout and real view of the printed accelerometer are shown in Figure 3a,b [21]. The accelerometer consists of a suspended square-shaped PET membrane, with a thickness *t*, of 140 µm, clamped by four crab-leg beams to a fixed support. The readout strategy is based on a set of strain gauges connected in series and printed on the four crab-leg beams. A proof mass of 0.550 × 10^−3^ kg has been attached in the center of the square-shaped plate with the aim to fit the frequency response of the accelerometer to the requirements of the applications and to increase the device responsivity. The frequency response (Figure 3c) of the device along with its expected behavior, is in line with the design choice. In particular, a device responsivity of 9.4 mV/g and 41.0 mV/g has been estimated at 10 Hz and 35 Hz, respectively. The corresponding calibration diagrams are shown in Figure 3d,e, including the estimated uncertainty bands at the 1*σ* level. The latter are ±0.0068 g at 10 Hz and ±0.0124 g at 35Hz.

### 3.3. An All-InkJet Printed Bending Actuator with Embedded Sensing Feature and an Electromagnetic Driving Mechanism

**Figure 4:**
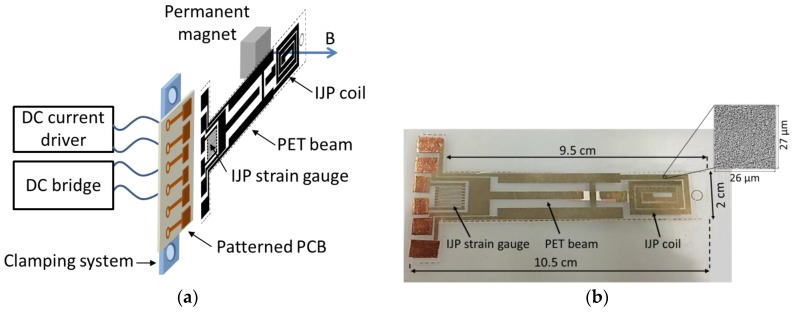

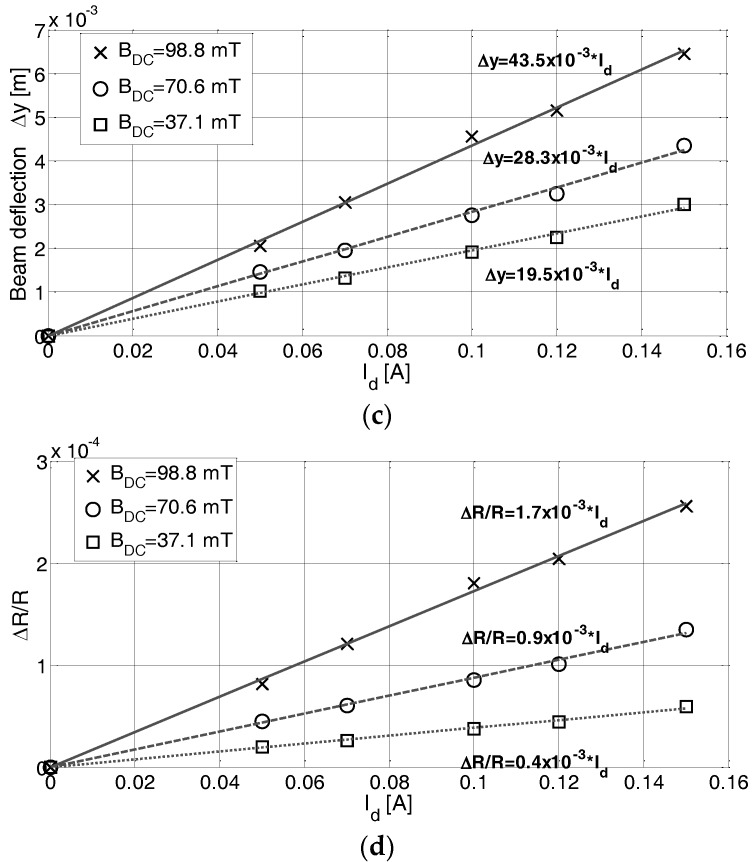
Schematization (**a**) and real view (**b**) of the IJP actuator developed at the SensorLab@DIEEI of the University of Catania, Italy. The beam deflection and the relative resistance change of the IJP strain gauge as a function of the driving current and the magnetic field are given in frames (**c**,**d**), respectively.

Despite the high performances of high voltage-operated actuators, the need for low-voltage, easy-to-use, and inexpensive actuators emerges in many applications, e.g., where disposable devices are required. The schematization and real view of an inkjet-printed actuator are shown in Figure 4a,b [23]. The device consists of a conductive coil printed on a flexible PET beam, with a thickness of 140 µm, and an external permanent magnet. The bending mechanism is actuated through the magnetic force generated by the interaction between the magnetic field and a DC current in the coil. A resistive readout strategy has been implemented on the beam using an inkjet-printed strain gauge. The main task of the printed strain sensor is to monitor the beam deflection; this is also necessary for the realization of a closed loop beam positioning system [23]. The printing process adopted to realize both the coil and the resistive sensor on the PET substrate uses a low-cost EPSON piezo inkjet printer and a silver nano-particle solution “Metalon^®^ JS-B15P” by Novacentrix. Figure 4c,d show the beam deflection and the relative resistive response of the device as a function of the driving current I_d_ and the target magnetic field B_DC_. As expected, the beam deflection linearly increases with the applied current, while the resistive output can be used to implement a deflection feedback mechanism.

### 3.4. A Low-Cost Snap-Through Buckling Inkjet Printed Device for Vibrational Energy Harvesting

In the following, a cheap bistable device in a snap-through-buckling (STB) configuration is illustrated; the device converts low-frequency mechanical vibrations into electrical energy [42]. Bistable resonators are known to provide performance far better than linear resonators when the vibrations occur in a wide range of frequency (rather than at a single frequency). The harvester was realized by exploiting a commercial office inkjet printer to print interdigitated (IDT) electrodes of a silver nano-particle solution (Metalon^®^ JS-B15P by Novacentrix) onto a PET substrate, coupled with a simple screen printing process for the realization of the piezoelectric layer. After deposition, the PZT layer was poled by applying an electrical field of about 2MV/m at a temperature of about 100 °C for 10 min. The IDT fingers have width, length, and thickness of 140 µm, 6 mm, and 200 nm, respectively, while the track spacing is 150 µm. A 1 mm pre-compression is applied along the *Y* axis of the beam in order to achieve the snap-through-buckling (STB) configuration.

**Figure 5:**
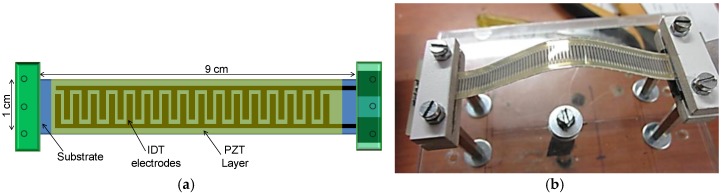
(**a**) Schematization and (**b**) real view of the STB harvester developed at the SensorLab@DIEEI of the University of Catania, Italy.

**Figure 6:**
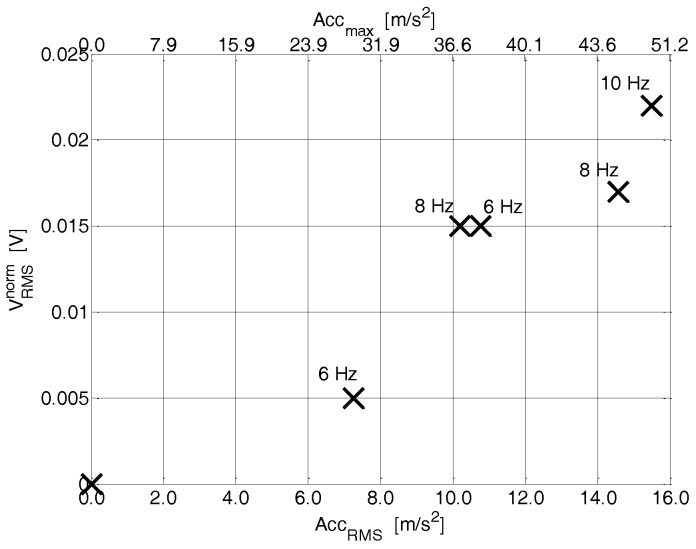
Normalized root mean square value of the output voltage, for different accelerations applied to the beam, in case of pre-compression ΔY = 1

The device (Figure 5), thanks to its nonlinear structure, exhibits rapid switching and large displacements, both of which are crucial in power conversion and an enhanced (when compared to a linear resonant device) power in the response. The root mean square value of the output voltage, for different accelerations applied to the beam, is shown in Figure 6. This result, as expected due the nonlinear structure of the system, demonstrates advantages with respect to traditional linear devices in terms of both the voltage generated and the operating frequency bandwidth [42].

## 4. Concluding Remarks

The need for the rapid prototyping of transducers requires setting benchmarks between different available technologies.

Which is the most suitable fabrication process for printed sensors? Can a sensor be fully realized by direct printing techniques? When can the term “rapid prototyping” be correctly used in the framework of printed sensors? In this paper, a comparison between screen printing, inkjet printing and hybrid processes is addressed, with the aim of developing criteria useful for the correct selection of the most appropriate technology depending on the development context and application area.

Screen printing can be successfully used for large-scale production. The need for masks, waste of materials, and the physical interaction between the printing system and the substrate are the main drawbacks of screen printing techniques. Thus, it can be affirmed that screen printing cannot be considered suitable for the rapid prototyping of lab-scale prototypes.

Short production time, low-cost, and material saving, as well as high spatial resolution and good reproducibility can be listed as main advantages of inkjet printing. In contrast with screen printing techniques, well defined, and complex topologies can be drawn without the need of patterning techniques and a waste of materials. Moreover, the contactless operation of the printing head allows for the use of such technique with different kind of substrates. Minor drawbacks (in inkjet printing) are related to the cost of inks; when office printers are used, the main restrictions are predicated by the ink compatibility. With all this said, inkjet printing appears to be the best solution for the rapid prototyping of electronic devices, including sensors, by implementing a real direct printing approach. Although interesting results have been obtained through a hybrid approach, as already outlined, such processes do not allow for the real implementation of rapid prototyping due to screen printing requirements.

A comparison between direct printing technologies is given in Table 1. The presented benchmarks emphasize that low-cost inkjet printing is particularly convenient for the rapid prototyping of customized simple components, especially for research purposes, proofs-of-concept, and applications requiring disposable devices.

In an era of small-cheap-disposable sensors, printed sensors seem destined to play a central role in sensing/processing applications. Setting aside the obvious military applications, we can envision a number of potential commercial applications. Many of these applications would involve mounting the sensor onto very small platforms, e.g., mini (insect-sized)-drones, and small unmanned vehicles. The sensors could be used in applications like oceanographic magnetic- and electric field-based surveying (topographical mapping, seeking oil/ore deposits, tracking the migration/movement of species), and aerial monitoring applications ranging from observing highway traffic to studying the terrestrial electromagnetic field. Other realizations of printed devices could serve as patch antennas on small UAVs, sensors to monitor parking in a city, pressure sensors on assorted surfaces, e.g., touch-screens, and sensors to monitor ambient effects, e.g., humidity, temperature, and water salinity. Using existing technology, the sensor can be paired with its own micro-scale energy harvester, thereby rendering it battery-less. Hence, some signal processing/conditioning could be done on board the sensor, and there would be power available to transmit some information via wireless nodes. Clearly, there are many more applications than listed above, with the number of applications constrained solely by the limits of our imagination. All-in-all, however, the future for printed sensors is quite bright.

## Figures and Tables

**Table 1:** Benchmark between printing techniques.

Technology	Advantages	Drawbacks
**Screen printing**	High printing speed Availability of many materials Realization of complex & multilayer devices	Need for masks Low spatial resolution Time consuming
**Low-cost inkjet piezoelectric printers**	Good spatial resolution (e.g., 5760 × 1440 dpi) Repeatability (~300 μm) Maskless Low-cost printing system Low-cost production	Unsuitable printing speed for mass production Unsuitable cost of inks for mass production Restricted kinds of conductive and functional materials
**Professional inkjet systems**	High spatial resolution Repeatability range [5 μm–25 μm] Maskless Compatibility with several materials Low-cost production	High cost printing system
**Mixedscreen printing & low-cost Inkjet printing**	Availability of many materials Good spatial resolution Repeatability (~300 μm)	Unsuitable printing speed for mass production Unsuitable cost of inks for mass production Need for masks Time consuming
